# Supplementary material for: Elementwise approach for simulating transcranial MRI-guided focused ultrasound thermal ablation
Source: Phys Rev Res. Author manuscript; Available in PMC 2021 Jun 22. (PMC8218657; doi:10.1103/physrevresearch.1.033205)
Supplement: Supplemental material [file NIHMS1593991-supplement-Supplemental_material.pdf]

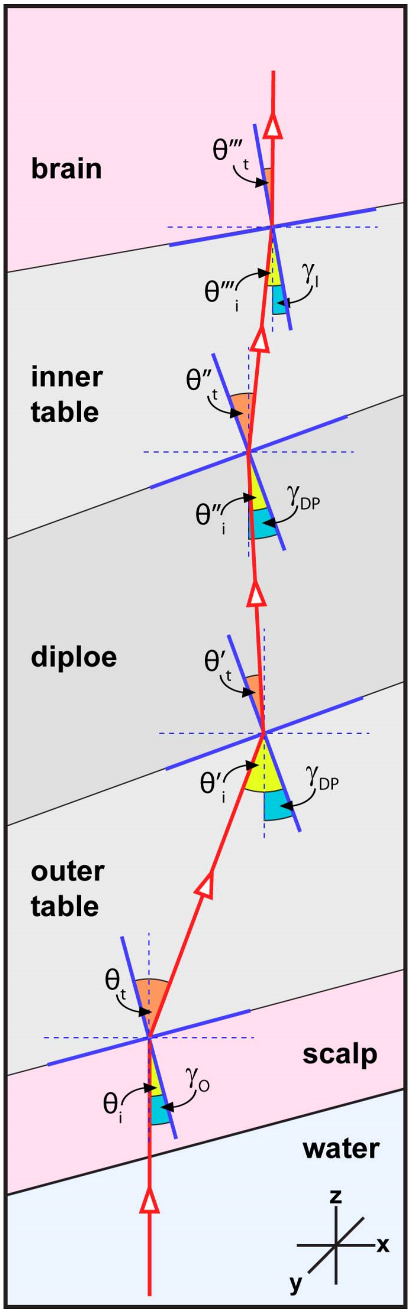

FIG. S1. Definitions of the angles used in the simplified model used to optimize the density/sound speed relationship. The incidence angles between the direction of ultrasound propagation (z) for each transducer element and the outer and inner skull surface and the diploe (γ_O, γ_I, and γ_{DP}) was obtained for each 44×44 x,y coordinate. We assumed that the incidence angles between the outer and inner tables and the diploe were the same.

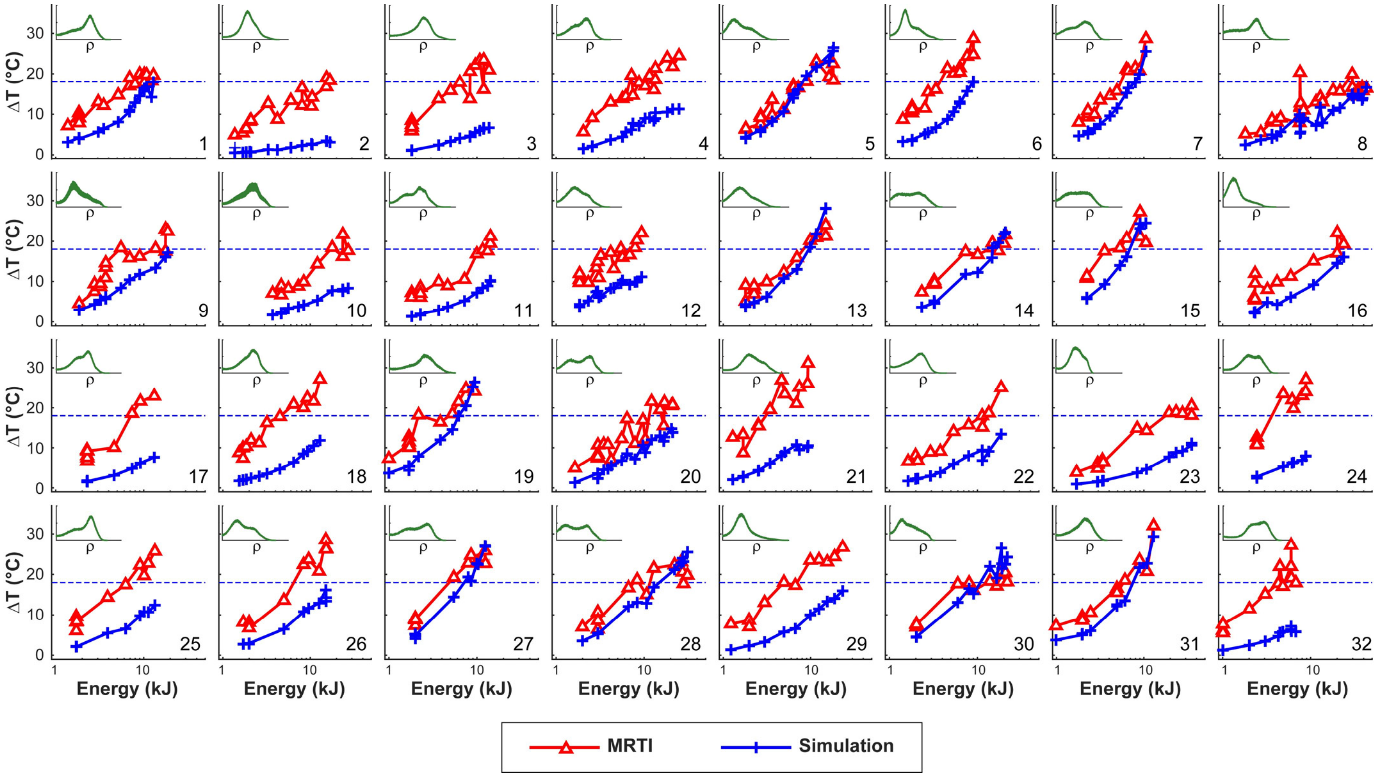

FIG. S2. Measured and simulated focal heating as a function of the applied acoustic energy for 32 patients using the attenuation/density and sound speed/density relationships found by Pichardo et al. [15]. The horizontal dotted lines indicate an absolute temperature of 55°C, a rough estimate for thermal necrosis.

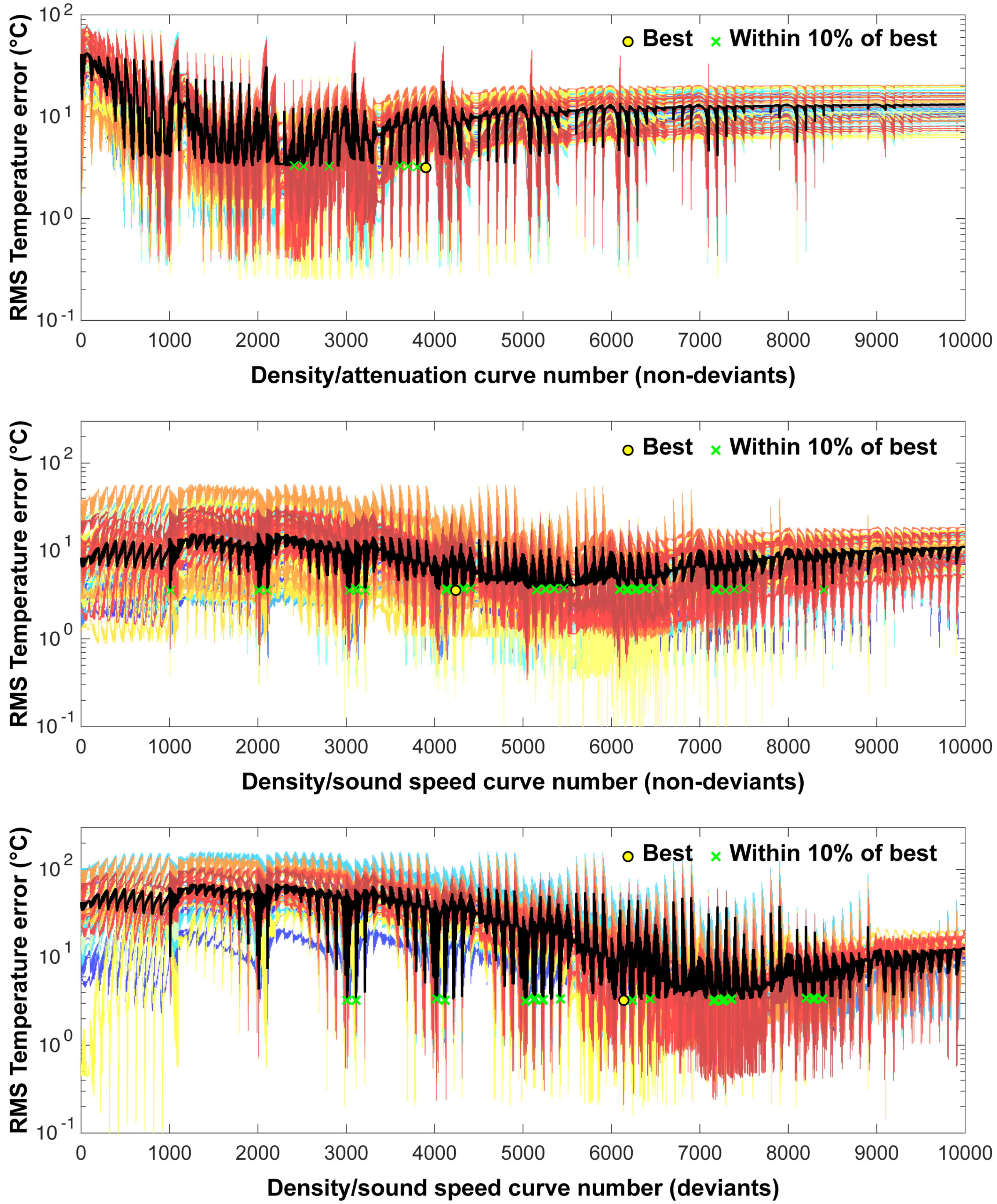

FIG. S3. Root-mean-squared (RMS) difference between simulations and measurements for the 10,000 different relationships between skull density and attenuation/sound speed that were examined in the optimization study. The thin colored lines plot the RMS error for each of the 72 patients. The thick black line is the error when all sonications from all patients are considered together. The curve number that produced the minimum error along with those within 10% of the minimum are indicated.

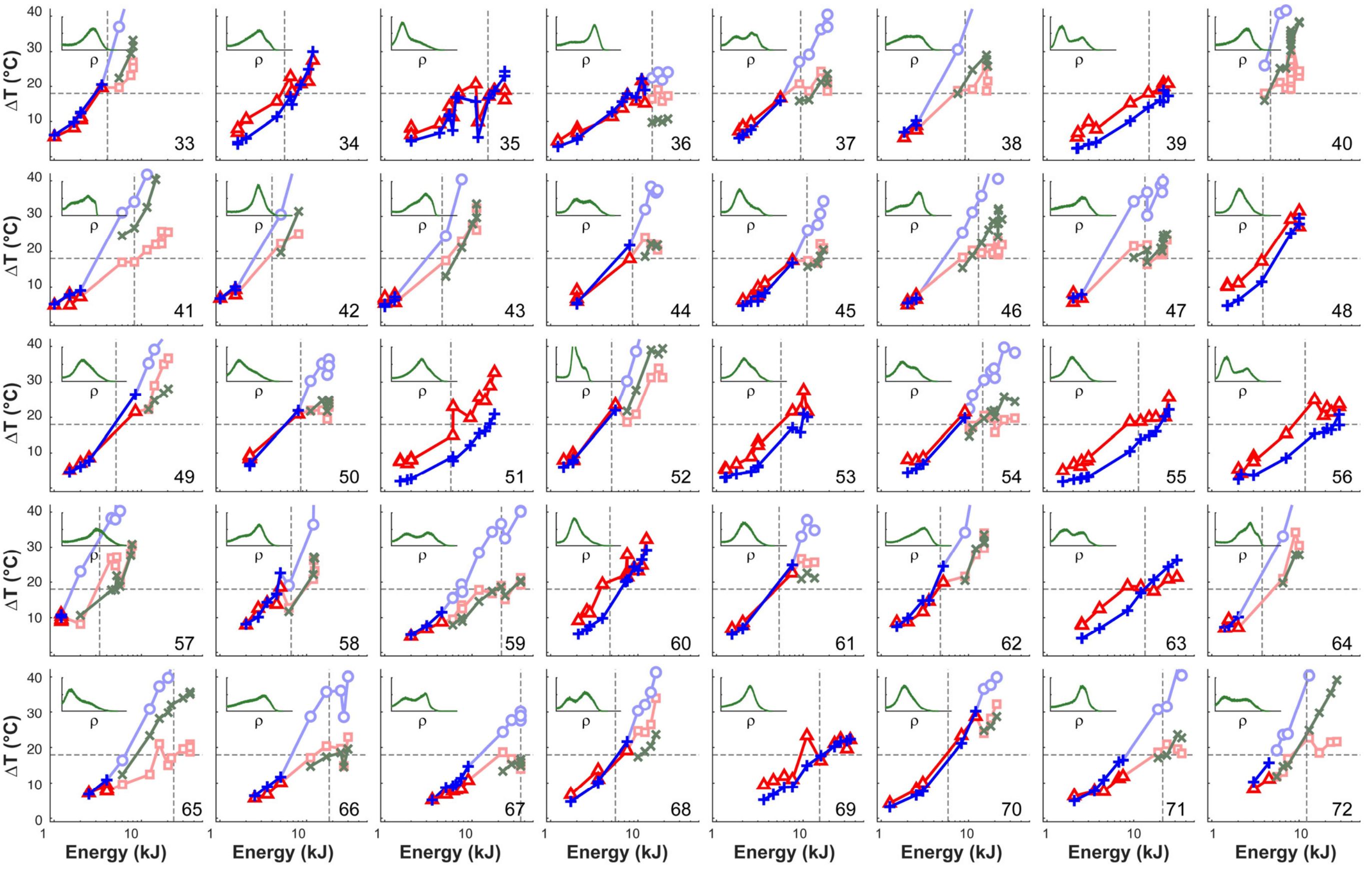

FIG. S4. Measured and simulated focal heating as a function of the applied acoustic energy for 40 patients using the attenuation/density and sound speed/density relationships optimized in patients 1-32. The horizontal dotted lines indicate an absolute temperature of 55°C, a rough estimate for thermal necrosis. The insets show histograms of skull density (x-axis: 1290-3500 kg/m³)

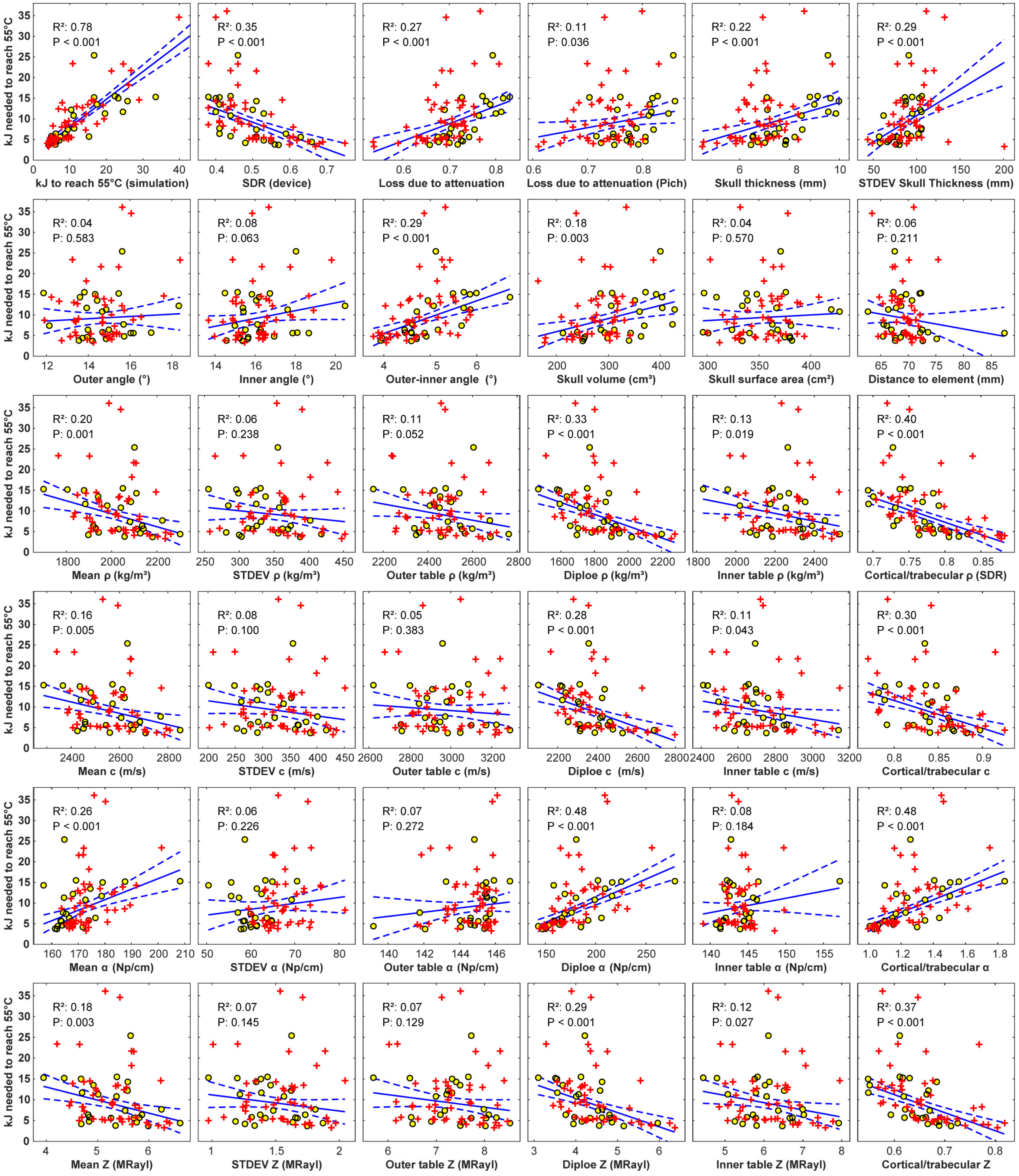

FIG. S5. Investigating predictors of the acoustic energy needed to achieve a focal temperature of 55°C. Features were extracted on a per-element basis from the CT scans. Patients where deviations in heating vs. acoustic energy are indicated by "+" symbols; Results of robust linear regression are noted. In most cases, the three patients that required the highest energy were outliers. Results from the simulations and the "skull density ratio" (SDR) provided by the device manufacturer are also included.

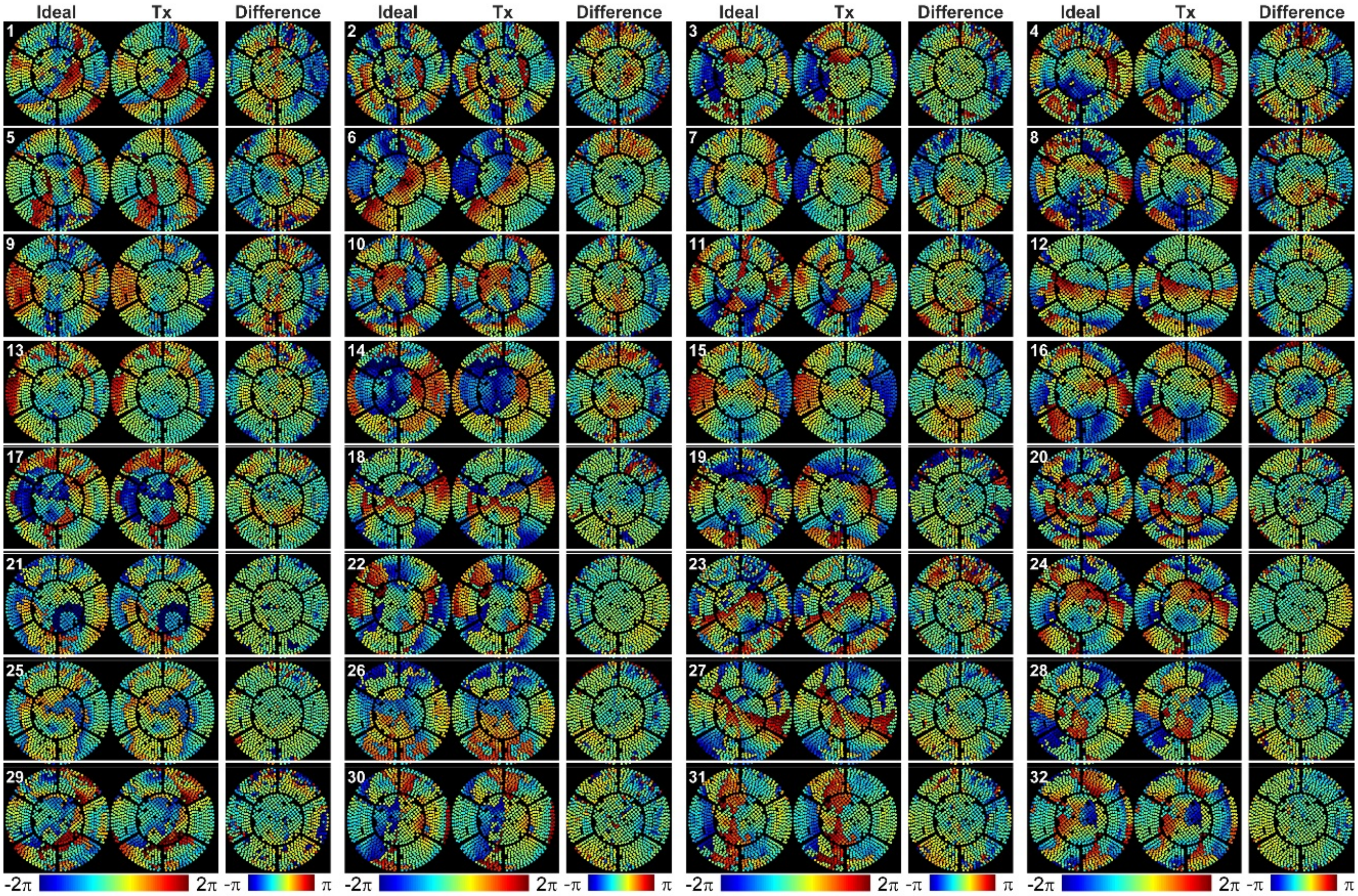

FIG. S6. Predicted vs. treatment phase corrections. Maps of the 1024-element transducer array showing the phase offsets used for aberration correction during the treatment, “ideal” corrections predicted by the simulations, and their difference, for 32 patients. The simulation produced phase offsets that were similar to those used in the treatments in every patient for most of the elements.

Table S1. Patient, treatment, and CT information for the 40 patients in the validation study.

Patient			Treatment						CT Scan			
N	Age	SDR	# son.	Power (W)	Duration (s)	Acoustic energy (kJ)			Vendor	Kernel	keV	Voxel (mm)
						Max.	Total	To reach 55°C				
33	73M	0.75	11	94-744	11-13	8.0	43.4	4.1	GE	BONE+	120	0.49×0.49×0.63
34	67F	0.63	9	145-986	12-13	11.8	54.0	5.6	GE	BONE+	120	0.43×0.43×0.63
35	85F	0.41	16	185-987	10-36	23.7	144.6	15.2	SIEMENS	H60s	120	0.43×0.43×1.00
36	72M	0.58	14	140-893	10-32	22.1	131.0	14.6	GE	BONE+	120	0.49×0.49×0.63
37	68M	0.46	10	184-1018	12-20	19.1	88.8	9.4	SIEMENS	H60s	130	0.44×0.44×1.00
38	81M	0.41	8	184-1112	12-18	16.6	74.3	9.1	SIEMENS	H60s	120	0.41×0.41×1.00
39	72F	0.40	10	184-1241	12-23	25.0	124.3	15.0	SIEMENS	Hr59h\2	120	0.45×0.45×1.00
40	67M	0.55	15	369-840	12-13	10.1	117.6	4.7	SIEMENS	H60s	120	0.47×0.47×1.00
41	74M	0.53	11	91-1118	12-19	20.2	100.8	8.3	Philips	C	120	0.49×0.49×1.00
42	76F	0.68	5	93-740	12-12	8.1	17.2	4.0	SIEMENS	H60s	120	0.43×0.43×1.00
43	66M	0.65	12	91-1068	11-13	11.3	53.2	4.5	GE	BONE+	140	0.49×0.49×0.63
44	75M	0.49	8	186-1114	12-16	16.8	73.2	8.7	SIEMENS	H60s	100	0.50×0.50×1.00
45	60F	0.55	10	187-1131	12-18	17.2	80.7	11.1	GE	BONE+	120	0.48×0.48×1.00
46	78M	0.44	17	184-1297	12-23	24.2	216.4	13.0	SIEMENS	H60s	120	0.49×0.49×1.00
47	72M	0.42	12	186-1128	12-28	24.1	176.8	13.5	SIEMENS	H60s	120	0.50×0.50×1.00
48	68F	0.56	7	140-745	12-14	10.0	36.9	3.8	SIEMENS	H60s	120	0.46×0.46×1.00
49	73F	0.50	8	139-1130	12-19	20.3	79.0	5.1	SIEMENS	H60s	120	0.41×0.41×1.00
50	89F	0.38	10	231-1122	10-22	18.1	96.5	8.6	SIEMENS	H60s	120	0.41×0.41×1.00
51	70F	0.51	12	138-1126	8-17	18.1	90.9	5.7	TOSHIBA	FC30	120	0.40×0.40×1.00
52	68F	0.51	10	138-1109	11-18	19.2	79.9	5.0	Philips	C	120	0.45×0.45×1.00
53	75F	0.51	10	138-1116	10-15	11.2	51.0	5.6	SIEMENS	H60s	120	0.45×0.45×1.00
54	84M	0.44	12	186-1124	12-37	33.9	170.2	14.5	SIEMENS	H60s	120	0.46×0.46×1.00
55	73F	0.53	12	137-1285	12-21	25.5	117.1	11.3	SIEMENS	H60s	120	0.47×0.47×1.00
56	73F	0.40	12	184-1134	12-28	28.9	154.2	11.7	SIEMENS	Hr59h\2	120	0.45×0.45×1.00
57	76M	0.67	12	137-782	10-12	7.8	43.9	3.3	TOSHIBA	FC30	120	0.47×0.47×1.00
58	68M	0.48	11	149-846	9-18	12.4	60.5	12.4	SIEMENS	H60s	120	0.47×0.47×1.00
59	79M	0.44	12	187-1167	11-32	36.1	177.5	21.7	SIEMENS	H60s	100	0.46×0.46×1.00
60	79F	0.53	11	190-1139	11-12	12.5	76.5	4.8	SIEMENS	H60s	120	0.46×0.46×1.00
61	66F	0.55	7	140-1039	11-19	13.6	47.3	5.3	SIEMENS	H60s	120	0.48×0.48×1.00
62	65M	0.62	10	141-1146	11-14	15.1	81.7	4.8	GE	BONE+	120	0.49×0.49×0.63
63	87M	0.41	8	235-1172	11-30	31.5	103.3	13.5	SIEMENS	H60s	120	0.47×0.47×1.00
64	83M	0.60	6	141-938	11-12	10.0	30.6	3.8	Philips	D	120	0.46×0.46×1.00
65	92F	0.38	15	239-1144	7-36	36.1	236.0	23.4	SIEMENS	H60s	120	0.45×0.45×1.00
66	87M	0.46	8	236-1126	11-44	30.0	120.8	18.2	GE	BONE+	120	0.49×0.49×0.63
67	74M	0.43	12	331-1101	11-34	36.1	232.7	36.1	SIEMENS	H60s	120	0.45×0.45×1.00
68	73M	0.53	8	190-1122	11-16	16.1	68.5	5.5	SIEMENS	Hr59h\2	120	0.53×0.53×1.00
69	79F	0.44	10	332-1043	11-40	35.0	165.9	15.5	SIEMENS	H60s	120	0.41×0.41×1.00
70	89F	0.51	8	140-1129	12-20	21.0	81.2	5.8	SIEMENS	H60s	120	0.45×0.45×1.00
71	89M	0.51	9	191-1145	12-37	35.8	135.4	21.6	SIEMENS	H60s	120	0.47×0.47×1.00
72	69M	0.43	9	281-1136	11-25	27.0	107.2	12.2	SIEMENS	H60s	120	0.47×0.47×1.00

SDR: “skull density ratio”

Supplemental Methods

In the exploration the ability of different skull-derived factors to predict the energy needed to reach 55°C and to estimate the transmission coefficient in the simplified sound speed model, we used the following formulae. The simulation (*xyz*) space consisted of 44×44×492 elements. The density of the skull was interpolated into this space for each element. For each *x* and *y* coordinate of these elemental volumes, we calculated the following factors:

Mean skull density, sound speed, attenuation, impedance:

$$\overline{\rho_z} = \frac{1}{z_b - z_a}\sum_{z=z_a}^{z=z_b} \rho(z),\ \ \overline{c_z} = \frac{1}{z_b - z_a}\sum_{z=z_a}^{z=z_b} c(\rho(z)),\ \ \overline{\alpha_z} = \frac{1}{z_b - z_a}\sum_{z=z_a}^{z=z_b} \alpha(\rho(z)),\ \ \overline{Z_z} = \frac{1}{z_b - z_a}\sum_{z=z_a}^{z=z_b} \rho(z)\cdot c(\rho(z))$$

Where a and b are the coordinates of the outer and inner surface, respectively. We also calculated the standard deviation of the acoustic properties for the points between z_a and z_b.

Skull density, sound speed, attenuation at the outer table:

$$\overline{\rho_{OT}} = \frac{1}{3}\sum_{z=z_{OT}-1}^{z=z_{OT}+1} \rho(z),\quad \overline{c_{OT}} = \frac{1}{3}\sum_{z=z_{OT}-1}^{z=z_{OT}+1} c(\rho(z)),\quad \overline{\alpha_{OT}} = \frac{1}{3}\sum_{z=z_{OT}-1}^{z=z_{OT}+1} \alpha(\rho(z)),$$

$$\overline{Z_{OT}} = \frac{1}{3}\sum_{z=z_{OT}-1}^{z=z_{OT}+1} \rho(z)\cdot c(\rho(z))$$

These values at the inner table and the diploe were found in the same way.

Loss due to skull attenuation:

$$L = \exp\left(-\sum_{z=z_a}^{z=z_b} \alpha(\rho(z))\cdot \Delta z\right)$$

Trabecular/cortical ratio:

$$\rho_{ratio} = \frac{0.5\cdot(\overline{\rho_{IT}} + \overline{\rho_{OT}})}{\overline{\rho_{DP}}}\quad c_{ratio} = \frac{0.5\cdot(\overline{c_{IT}} + \overline{c_{OT}})}{\overline{c_{DP}}}\quad \alpha_{ratio} = \frac{0.5\cdot(\overline{\alpha_{IT}} + \overline{\alpha_{OT}})}{\overline{\alpha_{DP}}}\quad Z_{ratio} = \frac{0.5\cdot(\overline{Z_{IT}} + \overline{Z_{OT}})}{\overline{Z_{DP}}}$$

The mean value of each metric was calculated for each transducer element. Results presented are the means or standard deviations over all the elements for each patient.
